# The relationship between dietary lipophilic index and load with depression, anxiety, and stress symptoms

**DOI:** 10.1186/s12888-023-05161-5

**Published:** 2023-09-27

**Authors:** Mahdieh Barzegaran, Shima Jazayeri, Jamileh Abolghasemi, Mahdieh Hosseinzadeh, Seyedeh Fatemeh Fatemi, Masoud Mirzaei, Amin Salehi-Abargouei

**Affiliations:** 1 Department of Nutrition , School of Public Health, Iran University of Medical Sciences, Tehran, Iran; 2 Department of Biostatistics, School of Public Health, Iran University of Medical Sciences, Tehran, Iran; 3 Department of Nutrition, School of Public Health, Shahid Sadoughi University of Medical Sciences, Yazd, Iran; 4 Yazd Cardiovascular Research Center, Non-communicable Diseases Research Institute, Shahid Sadoughi University of Medical Sciences, Yazd, Iran; 5 Research Center for Food Hygiene and Safety, School of Public Health , Shahid Sadoughi University of Medical Sciences, Yazd, Iran; 6 Department of Nutrition, Faculty of Medicine, Mashhad University of Medical Sciences, Mashhad, Iran

**Keywords:** Lipophilic index, Lipophilic load, Depression, Anxiety, Stress

## Abstract

**Background:**

Dietary fatty acids can affect brain health by modifying neuronal membrane fluidity. Dietary lipophilic index (LI) and load (LL) may be related to cell membrane fluidity. This study aimed to determine the relationship between dietary LI and LL with symptoms of depression, anxiety and stress.

**Methods:**

In this cross-sectional study, taken from the YaHS (Yazd Health Study) population-based cohort, the data of 2,982 individuals was extracted. Several questionnaires- a 178-item Food Frequency Questionnaire (FFQ), Depression, Anxiety and Stress Scale 21 (DASS 21), and International Physical Activity Questionnaire (IPAQ)- were used to obtain information on dietary intake, mental status, and physical activity, respectively. LI and LL were calculated using dietary intake and the melting point of each fatty acid.

**Results:**

The analysis was performed on 2982 individuals. The odds ratio of depression in the second tertile of dietary LI compared to the first tertile was 0.815 (95% CI 0.66–1.00, *P* = 0.051, P_trend_ = 0.017) and after adjusting confounders was 0.793 (95% CI 0.63–0.99, *P* = 0.043, P_trend_ = 0.011). Also, LL was related inversely with anxiety (0.771, 95% CI 0.63–0.93, *P* = 0.003) that after multiple regression, OR of anxiety was 0.762 (95% CI 0.53–1.07, *P* = 0.045). The odds of stress in the third tertile of LL was 1.064 but not statistically significant (95% CI 0.88–1.28, *P* = 0.729).

**Conclusion:**

This study showed an inverse association between dietary LI and depression symptoms. Anxiety and stress did not show a significant relationship with LI or LL.

**Supplementary Information:**

The online version contains supplementary material available at 10.1186/s12888-023-05161-5.

## Introduction

Mental disorders, especially depression and anxiety, are major health-related burden factors around the world, and after 2020, due to the Covid-19 pandemic, these disorders and their effects have become more serious [1]. Global statistics in 2017 showed that depression and anxiety affect 322 and 264 million people, respectively [2].

Various psychological, genetic, developmental, and biological factors may increase the risk of depression [3], and nutrition may be one factor [4]. These factors can lead to this disorder by affecting cell membrane fluidity—a key component and mediator in the progression of depression. Reduction of the membrane fluidity will disrupt the normal function of the cell, and the cell's sensitivity to injury and death will increase [5]. Many researches have examined the impact of fatty acids on depression, anxiety, and stress and reported different results. In a 2021 review study, Bozzatello et al. indicated that daily supplementation of 0.6–2.5 g of EPA^1^ and DHA^2^ reduced depressive symptoms in patients with MDD,^3^ bipolar disorder, and anorexia nervosa [6]. Another study mentioned supplementation of omega-3 could be as effective as antidepressants with minimal side effects [7]. The results of a meta-analysis in the same year showed that, contrary to popular belief about the protective effects of PUFAs^4^ Against depression and anxiety, long-chain omega-3 fatty acids may have inconsiderable or no effect on preventing symptoms of depression or anxiety [8]. Most of the studies conducted so far in the field of fatty acids have been on specific types of fatty acids (such as EPA and DHA) and only their quantities. At the same time, the diets of people contain several fatty acids, each of which has its quantity and quality and can have different effects on a person's health, and it is necessary to use a method to assess the quantity and quality of all fatty acids in people’s diet; The lipophilic index (LI) can bring us closer to this goal. In calculating this new index, the intake of most fatty acids and their melting points, which indicate the fluidity of fatty acids and their quality, are considered [9]. Melting points and quantity of dietary fats affect the lipid composition of membrane phospholipids and, consequently, membrane fluidity [10, 11]. Melting points increase with increasing chain length and level of saturation of fatty acid, leading to decreased cell membrane fluidity [12]. Previously, an increase in dietary LI and a consequent decrease in cell membrane fluidity have been studied in several studies and have shown positive or ineffective correlations. A cross-sectional survey of Soltani et al. Showed a consistent relationship between LI and BMI,^5^ WC,^6^ and SBP^7^ [13]. Also, in another study by Suara et al., LI was positively associated with general obesity and weight-to-height ratio, and abdominal obesity was significantly higher in the fifth quintile than in the first quintile of the LI [14]. A cohort study showed that an increase in dietary LI was related to an increased risk of CHD^8^ after menopause [15]. Toledo et al., also found that an increase in dietary LI was linked to an increase in plasma TG,^9^ a higher ratio of LDL^10^ to HDL,^11^ and risk of myocardial infarction (in the highest quintile of the dietary LI) [16]. On the other hand, Sluijs et al. In Italy demonstrated that dietary lipophilic load (LL) and index were not associated with CHD and ischemic stroke [17].

On the other hand, previous studies have reported relationships between depression and atherogenic indicators, metabolic syndrome and obesity. Beydoun et al. reported that atherogenic indices, such as a high ratio of LDL to HDL, are directly related to a faster increase in depressive symptoms among women [18]. Also, in another study, more depressive symptoms were associated with more HDL and TG disorders [19]. Also, people with MDD compared to the general population, have a 50% higher risk of developing obesity [20].

Considering the various consequences of depression, anxiety, and stress and the lack of a study to evaluate the association between dietary LI and LL with these psychological symptoms, this study aimed to examine whether a higher LI and LL is associated with higher depression, anxiety, and stress symptoms based on a population study in Yazd.

## Methods

### Study population and data collection

This cross-sectional study is a part of the YaHS.^12^ YaHS is a population-based cohort study that was started in September 2014-March 2016 for Yazd greater area, and information is collected every five years. Currently, information on 9962 people aged 20–70 in urban and rural regions of Yazd is available. This cohort aims to study the changes in the incidence of a range of chronic diseases and their risk factors in Yazd because Yazd is one of the provinces with a high incidence of chronic diseases. Two-stage cluster random sampling was used, and initially, 200 random clusters were chosen based on residential postal code. After arranging a meeting time, interviewers visited the selected individuals, and all participators affirmed informed consent. The ethical agreement of the cohort has been obtained from the ethics committee of Shahid Sadoughi University of Medical Sciences, Yazd, number IR.SSU.REC.1393.7341 and the ethics committee of Iran University of Medical Sciences approved the execution of the present study (number IR.IUMS.REC.1400.206). The cohort information includes family history of non-communicable diseases, personal and dietary habits, physical activity, medical history, mental health status, and anthropometric measurements. The validity and reliability of the questionnaires before the study were assessed by collecting information in a pilot of 50 participants and evaluating them by specialists in each scope. A panel of experts confirmed face validity, and Cronbach's alpha 0.89 was used to approve the reliability of the questionnaires. Other aspects of this cohort have been reported [21]. Inclusion criteria, which were the same as the cohort criteria, were: age 20–69 years during the interview and informed consent to participate in the study. Exclusion criteria related to the subject of this cross-sectional study were: unbalanced energy intake (less than 800 and more than 4000 kcal per day), mental illness except for depression and anxiety, continuous usage (at least once a week) of vitamin-mineral supplements with anti-inflammatory and antioxidant characteristics, pregnancy and constant smoking.

### Dietary assessments and dietary lipophilic index and load calculation

Trained interviewers measured each participant's food intake using a 178-item FFQ^13^; this questionnaire is the same 168-item questionnaire in the TLGS^14^ to which ten items of particular foods in Yazd province have been added. This FFQ had been reported as reliable and valid [21–23]. First, by searching the USDA^15^'s food composition tables, the most similar foods to questionnaire items were selected. The amount of water, energy, macronutrients, and micronutrients (including 32 fatty acids) was entered into Excel. Therefore, macronutrient and micronutrient amounts per 100 g were obtained from 178 items. Then, by formulating Excel for each nutrient received by one participant, based on the available amounts of that nutrient in 100 g of 178 food items and generalizing that participant's formula to the whole sample, the amounts of fatty acids and other nutrients of all individuals were calculated. The melting points of 32 USDA fatty acids were searched and selected on the Lipid Bank database [24] (Additional file 1). From the cases with two or more melting points, their mean and the similar isoforms, the most famous ones, which are high consumption, were selected. It should be noted that, in general, out of 49 fatty acids, 37 cases of melting point have been reported in the lipid bank; of these, 32 cases are available in the USDA. Items that are unavailable cause negligible peaks in the chromatogram; Hence, melting point information of about 98% of fatty acids is used [13]. LI and LL can be calculated according to the following formulas:

$$\mathrm{DietaryLI}=\frac{{\sum }_{\mathrm{k}}^{\mathrm{i}}[\mathrm{fattyacid}\left(\mathrm{g}\right)\mathrm{i}\times \mathrm{meltingpoint}\left(\mathrm{^\circ{\rm C} }\right)\mathrm{i}]}{{\sum }_{\mathrm{k}}^{\mathrm{i}}\mathrm{fattyacid}\left(\mathrm{g}\right)\mathrm{i}}$$

$$\mathrm{DietaryLL}={\sum }_{\mathrm{k}}^{\mathrm{i}}[\mathrm{fattyacid}\left(\mathrm{g}\right)\mathrm{i}\times \mathrm{meltingpoint}\left(\mathrm{^\circ{\rm C} }\right)\mathrm{i}]$$

### Measurement of anthropometric variables and physical activity

The participants' weights using Omron BF511 portable digital scale and body analyzer (Omron Inc. Nagoya, Japan) with minimum clothing and accuracy of 100 g and height using a tape measure on a straight wall to the nearest centimeter and without shoes were measured. WC^16^ and HC^17^ were measured with a tape measure and an accuracy of 0.5 cm. Physical activity was evaluated with the short form of the IPAQ^18^ [25]. IPAQ has been good internal consistency (Cronbach's alpha 0.7) and test retest reliability (Spearman Brown correlation coefficient 0.9) [26].

### Psychological health assessment

Depression, anxiety, and stress were obtained by the DASS21^19^ that had been acceptable internal consistency (Cronbach's alpha 0.94), good reliability and very good convergent validity [27] This 21-item scale has seven questions for each subscale and is designed to diagnose and screen for symptoms of depression, anxiety, and stress over the past week. The scoring method is derived from a 4-point Likert scale, and each item is rated from 0, which means "did not apply to me at all" to 3 ", applied to me very much or most of the time”. Each subscale can have a score of 0–21, so by increasing the score of each subscale, the intensity of its (depression, anxiety, and stress) increases. For the final ranking, the values ​​obtained from each person had to be multiplied by 2, and the sum of the scores of ≥ 10, ≥ 8, and ≥ 15 indicates depression, anxiety, and stress, respectively.

### Statistical analysis

Data analyses were done using Statistical Package for Social Science (SPSS Inc., Chicago IL. Version 22.0). In the case of parametric test presuppositions, the comparison of participants’ general characteristics was made by independent t-tests and one-way analysis of variance (ANOVA) or to check the correlation between quantitative variables was applied Pearson correlation coefficient and in the case of non-parametric test presuppositions, were used Mann–Whitney, Kruskal–Wallis tests and Spearman correlation coefficient. The relationships between qualitative variables were examined with Chi-square. We used simple or multivariate binary regression analysis and odds ratio to present the final results. *P* < 0.05 was considered statistically significant.

## Results

### Participant’s characteristics

The details of the participants' general characteristics (*n* = 2982) have been presented in Table 1. They were generally male (53.5%), aged 40–49 years (21.4%) and married (84.3%). According to the cut points of the DASS21 questionnaire, 25.7%, 28.5%, and 30.7% of participants had significant symptoms of depression, anxiety, and stress, respectively.

**Table 1:** The comparison of characteristics by tertiles of dietary LI

Characteristics	(*N* = 2982)	LI tertiles			*P*-value^*^
		**1st tertile (*N* = 992)**	**2nd tertile** **(*N* = 994)**	**3rd tertile** **(*N* = 996)**	
Dietary LI(ºC)^a^	27.7(2.9)	25.2(2.11)^A^	27.7(0.94)^B^	29.6(1.53)^C^	< 0.001
Dietary LL(ºC*g)^a^	1842.3(1079.2)	1817.3(1115.5)^A^	1759.1(944.0)^B^	1944.5(1144.1)^C^	< 0.001
**Demographic variables, n (%)**					
Sex					
Male	1587(53.5)	525(17.7)	518(17.5)	544(18.4)	0.540
Female	1377(46.5)	464(15.7)	467(15.8)	446(15.0)	
Age (years)					
20–29	631(21.3)	217(7.3)	207(7.0)	207(7.0)	0.549
30–39	618(20.9)	196(6.6)	211(7.1)	211(7.1)	
40–49	634(21.4)	214(7.2)	199(6.7)	221(7.5)	
50–59	539(18.2)	167(5.6)	197(6.6)	175(5.9)	
60–69	542(18.3)	196(6.6)	172(5.8)	174(5.9)	
Education level					
Secondary school and lower	1539(52.1)	531(18.0)	500(16.9)	508(17.2)	0.080
Diploma and Graduate diploma	913(30.9)	295(10.0)	303(10.3)	315(10.7)	
Bachelor’s Masters and PhD	501(17.0)	157(5.3)	181(6.1)	163(5.5)	
Smoking					
Yes	78(2.6)	20(0.7)	35(1.2)	23(0.8)	0.288
No	2843(95.3)	952(31.9)	939(315)	952(31.9)	
Ex_smoking	61(2.0)	20(0.7)	20(0.7)	21(0.7)	
Marital status					
Married	2487(84.3)	828(28.1)	824(27.9)	835(28.3)	0.874
Single	372(12.6)	121(4.1)	128(4.3)	123(4.2)	
Widowed or Divorced	91(31)	33(1.1)	32(1.1)	26(0.9)	
Occupation					
Unemployed	583(20.0)	187(6.4)	204(7.0)	192(6.6)	0.539
Government-employed	1397(48.0)	454(15.6)	472(16.2)	471(16.2)	
Manual worker	102(3.5)	30(1.0)	29(1.0)	43(1.5)	
Self-employed	826(28.4)	285(9.8)	270(9.3)	271(9.3)	
**Anthropometric variables**					
Weight (kg)	72.63 (14.2)	72.53(14.2)	72.70(14.0)	72.66(14.4)	0.964
Height (cm)	164.76 (10.0)	164.44(10.1)	164.78(9.8)	165.06(10.2)	0.401
BMI (kg/m2)	26.78 (4.8)	26.83(4.8)	26.81(4.8)	26.71(4.9)	0.845
WC (cm)	93.34 (12.9)	93.61(12.7)	93.04(12.9)	93.37(13.1)	0.616
HC (cm)	101.36 (11.4)	101.32(11.6)	101.29(11.3)	101.45(11.3)	0.949
Body fat(%)	31.33(10.8)	31.44(10.8)	31.51(10.6)	31.04(11.0)	0.586
**Dietary factors**					
Carbohydrates (g)	280.54(126.4)	301.16(135.4)^A^	278.33(113)^B^	266.19(128.8)^C^	< 0.001
Protein (g)	123.38(68.7)	120.09(75.3)	125.37(63.7)	123.69(67.6)	0.232
Fat (g)	76.69(45.8)	83.65(51.5)^A^	72.58(38.7)^BD^	73.81(44.2)^CD^	< 0.001
Total energy(kcal)	2148.29(973.3)	2275.98(1128.2)^A^	2119.42(821.1)^BD^	2029.67(992.6)^CD^	< 0.001
SFA(g)	24.3(14.1)	23.3(13.2)^AB^	23.1(12.5)^BC^	26.6(15.8)^D^	< 0.001
MUFA(g)	27.2(17.9)	30.9(21.5)^A^	25.9(14.3)^BC^	25.5(15.9)^CD^	< 0.001
PUFA(g)	16.1(10.1)	20.7(13.9)^A^	15.3(8.7)^B^	13.0(7.8)^C^	< 0.001
TFA(g)	0.5(0.4)	0.5(0.5)^AB^	0.5(0.4)^BC^	0.4(0.3)^D^	< 0.001
W_3_ PUFA(g)	0.5(0.3)	0.6(0.4)^A^	0.5(0.2)^B^	0.4(0.2)^C^	< 0.001
PA (MET-h/week)^a^	657.00(971.0)	619.00(929.0)	670.37(974.5)	719.87(1003.6)	0.449
**Psychological status, n (%)**					
Depression					
Yes	767(25.7)	262(8.8)	225(7.5)	280(9.4)	0.017
No	2215(74.3)	730(24.5)	769(25.8)	716(24.0)	
Anxiety					
Yes	850(28.5)	295(9.9)	256(8.6)	299(10.0)	0.062
No	2132(71.5)	697(23.4)	738(24.8)	697(23.4)	
stress					
Yes	915(30.7)	315(10.6)	298(10.0)	302(10.1)	0.661
No	2067(69.3)	677(22.7)	696(23.3)	694(23.3)	

*LI* Lipophilic index, *LL* Lipophilic load, *BMI* Body mass index, *WC* Waist circumference, *HC* Hip circumference MET metabolic equivalent, *SFA* Saturated fatty acid, *MUFA* Mono saturated fatty acid, *PUFA* Poly saturated fatty acid, *TFA* Trans fatty acid, *SD* Standard deviation, *IQR* Interquartile range, ^a^Median (IQR) of continuous variables, ^b^Mean (SD) of continuous variables. The Kruskall-Wallis test or ANOVA is used to obtain the results. ABCD Mann–Whitney test; The dissimilar letters between the two groups in each row indicate a significant difference between the two groups. *Significance level was considered as *p* < 0.05

### Associations between LI or LL and psychological status

Table 1 compares dietary components, anthropometric indices, and physical activity in different LI tertiles. Median LI and LL values for the total population were 27.7 (2.9) ºC and 1842.3 (1079.2) ºC*g. Although in the lowest tertile of LI, total energy, carbohydrate, and fat intake are higher than all other tertiles, the consumption of saturated fatty acids is lower, and vice versa. Anthropometric indices (such as BMI and body fat percentage) and physical activity did not show a statistically significant difference between tertiles.

The relationship between dietary LI and LL with symptoms of depression, anxiety, and stress is shown in Table 2. According to the classification of the DASS21 questionnaire, individuals were divided into two groups with and without symptoms of depression, anxiety, or stress. The results showed that the OR^20^ in the highest tertile of dietary LI compared to the lowest tertile for depression was 1.090 (95% CI^21^ 0.89–1.32, *P* = 0.394, P_trend_ = 0.017). This OR after adjusting for confounders was 1.109 (95% CI 0.89–1.37, *P* = 0.350, P_trend_ = 0.011). On the other hand, persons in the second tertile of LI had significantly lower odds of depression compared with subjects in the first tertile (0.815, 95% CI 0.66–1.00, *P* = 0.051, P_trend_ = 0.017) and OR after adjustment was 0.793 (95% CI 0.63–0.99, *P* = 0.043, P_trend_ = 0.011). A positive association between the third tertile of LI, compared to the first tertile, and anxiety was observed (1.014, 95% CI 0.83–1.22, *P* = 0.063), which after adjusting confounders, this association was eliminated and excluded among the more effective variables. On the other hand, the odds of anxiety in the third tertile of LL, compared to the first tertile was significantly lower (0.771, 95% CI 0.63–0.93, *P* = 0.003) and after multiple regression, OR of anxiety was 0.762 (95% CI 0.53–1.07, *P* = 0.045), Due to the large number of comparisons performed, it is better to consider the significance level up to 0.025, and therefore OR of anxiety after adjusting was not significant. By evaluating the consumption trend of different oils, it was observed that as the LL increases from the first to the third tertile, the consumption of hydrogenated vegetable oil increases (mean (SD^22^) 1st, 2nd and 3rd tertile 2.5 (4.8), 3.8 (7.5) and 6.9 (13.8) respectively). Other results showed no statistically significant difference.

**Table 2:** Odds ratio(95% CI) of psychological status according to tertiles of dietary lipophilic index and load

**Tertile cutoffs**	**1st tertile (*N* = 992)**	**2nd tertile (*N* = 994)**	**3rd tertile(*N* = 996)**	*P*-trend^*^
**lipophilic index**				
** Depression**				
Crude model	Ref	0.815(0.66–1.00)	1.090(0.89–1.32)	0.017
Adjusted model	Ref	0.793(0.63–0.79)	1.109(0.89–1.37)	0.011
**Anxiety**				
Crude model	Ref	0.820(0.67–0.99)	1.014(0.83–1.22)	0.063
Adjusted model^b^	Ref	0.808(0.64–1.00)	1.013(0.81–1.26)	0.080
**Stress**				
Crude model	Ref	0.920(0.76–1.11)	0.935(0.77–1.13)	0.661
Adjusted model^c^	Ref	0.955(0.77–1.18)	0.871(0.69–1.08)	0.457
**lipophilic load**				
**Depression**				
Crude model	Ref	0.822(0.67–1.00)	0.845(0.69–1.03)	0.112
Adjusted model^a^	Ref	0.925(0.72–1.18)	1.023(0.71–1.46)	0.676
**Anxiety**				
Crude model	Ref	0.736(0.60–0.89)	0.771(0.63–0.93)	0.003
Adjusted model^b^	Ref	0.736(0.57–0.93)	0.762(0.53–1.07)	0.045
**Stress**				
Crude model	Ref	0.991(0.81–1.20)	1.064(0.88–1.28)	0.729
Adjusted model^c^	Ref	1.145(0.90–1.44)	1.146(0.81–1.60)	0.527

*LI* Lipophilic index, *LL* Lipophilic load. The simple or multivariate binary regression analysis is used to obtain the results. ^a^Adjusted for age, sex, education level, marital status, smoking, occupation, hypertension/diabetes/hypercholesterolemia status, physical activity, total energy, body fat, and calcium supplementation ^b^Adjusted for age, sex, education level, marital status, occupation, hypertension/diabetes/hypercholesterolemia status, physical activity, total energy, body fat, and vitamin D supplementation ^c^Adjusted for age, sex, education level, marital status, hypertension/diabetes/hypercholesterolemia status, physical activity, body fat, folate, and vitamin D supplementation. *Significance level was considered as *p* < 0.02

## Discussion

In the present study, we demonstrated an inverse association between dietary LI and depression in the second tertile of LI compared to the first. The study by Liu et al. On the risk of CHD after menopause and that an increase in dietary LI was related to an increased risk of CHD [15]. Similarly, Toledo et al. Studied 1,627 people with a history of myocardial infarction and 1,627 healthy controls, who found that the highest quintile of the LI was directly related to increased odds of myocardial infarction [16]. Soltani et al., in 2020, who studied 504 adults, showed that an increase in LI was associated with increased BMI, WC and SBP [13]. Similar to this study, Suara et al., in a survey of 295 women, in 2020, demonstrated a positive relationship between LI and general obesity and overweight after adjusting confounders, and the odds of abdominal obesity was significantly higher in the fifth quintile than the first quintile of LI [14]. On the other hand, in our study, anxiety, and stress did not show a significant association with any of the LI or LL. This is consistent with some previous studies, such as the study by Sluijs et al., Which displayed that dietary LI was not associated with the odds of CHD and ischemic stroke [17].

Dietary LI and LL are new scales for estimating the quality along with the number of fatty acids consumed and are obtained by considering the melting points and the amount received by a wide range of fatty acids; reduction of these two indicators may indirectly indicate increased membrane fluidity. The premise of this study, which is the direct relationship between dietary LI or LL with psychological symptoms, can be explained by the fact that by consuming fatty acids with a lower melting point and the entrance of these fatty acids into the membrane phospholipids of neurons, membrane fluidity increased, and this improved brain cell function in several ways and prevented cell damage and death [5]. Improved membrane enzyme function, ion channel function [28], receptor and membrane lipid activity [29], reduced insulin resistance [30], prevented serotonin depletion, and inappropriate changes in the dopaminergic system [28] are all beneficial changes that could occur with increased membrane fluidity of neurons; It is recommended that future psychiatric biological studies test these potential mechanisms empirically. The final results in this study showed inverse or neutral associations that could be said different sources of fat and oil, depending on the potential interactions of nutrients as well as their different functional properties, can have complex effects on the body and brain health. On the other hand, psychiatric symptoms can be changed by factors other than nutrition, such as psychological, hereditary, evolutionary, and biological factors [3]. Also, participants may try to modify their diet after experiencing symptoms of depression, anxiety, and stress.

Regarding the important strengths of this study, it can be said that this is the first study that investigated the relationship between LI or LL in the diet and the symptoms of depression, anxiety and stress separately and in a large population of Iranian people. Another strength of this study is the up-to-dateness of food choices from USDA, which was tailored to the preferences of the people of Yazd.

### Limitations

Among the limitations of the present study, the following can be mentioned: 1) the Cross-sectional design of the study, which may not be very accurate in expressing the causal relationship; 2) the measurement error of the dietary assessment method 3) the self-report nature of the DASS21 questionnaire.

## Conclusion

The present cross-sectional study of 2982 individuals showed an inverse association between dietary LI and depression symptoms. Anxiety and stress did not show a significant relationship with LI or LL. More studies, particularly cohort research, are recommended to explain the causal relationship further. It is also better to use more accurate methods of measuring LI, including adipose tissue, RBC, and plasma LI.

### Supplementary Information

**Additional file 1:** **Appendix 1.** Melting points of different fatty acids and correlations between individual fatty acids and the LI.

## Data Availability

Because the data of our study was taken from the YaHS cohort, access to the data was subject to their permission. It will be available from the corresponding authors on reasonable requests.

## Abbreviations

LI: Lipophilic index
LL: Lipophilic load
YaHS: Yazd Health Study
FFQ: Food Frequency Questionnaire
DASS 21: Depression, Anxiety and Stress Scale 21
IPAQ: International Physical Activity Questionnaire
OR: Odds ratio
CI: Confidence interval
MDD: Major depressive disorder
EPA: Eicosapentaenoicacid
DHA: Docosahexaenoicacid
TLGS: Tehran Lipid and Glucose Study
USDA: United states department of agriculture
BMI: Body mass index
WC: Waist circumference
HC: Hip circumference
MET: Metabolic equivalent
SFA: Saturated fatty acid
MUFA: Mono saturated fatty acid
PUFA: Poly saturated fatty acid
TFA: Trans fatty acid
SD: Standard deviation
IQR: Interquartile range
CHD: Coronary heart disease
SBP: Systolic blood pressure

## Footnotes

1 Eicosapentaenoic acid.

2 Docosahexaenoic acid.

3 Major depressive disorder.

4 Polyunsaturated fatty acids.

5 Body Mass Index.

6 waist circumference.

7 systolic blood pressure.

8 Coronary Heart Disease.

9 Triglycerides.

10 Low Density Lipoprotein.

11 High Density Lipoprotein.

12 Yazd Health Study.

13 Food Frequency Questionnaire.

14 Tehran Lipid and Glucose Study.

15 United states department of agriculture.

16 Waist Circumference.

17 Hip Circumference.

18 International Physical Activity Questionnaire.

19 Depression, Anxiety, and Stress Scale21.

20 Odds Ratio.

21 Confidence Interval.

22 Standard Deviation.
