# Supplementary material for: The relationship between dietary lipophilic index and load with depression, anxiety, and stress symptoms
Source: BMC Psychiatry. 2023 Sep 27;23:703. doi: 10.1186/s12888-023-05161-5 (PMC10523600; doi:10.1186/s12888-023-05161-5)
Supplement: Supplementary file 1 — Additional file 1: Appendix 1. Melting points of different fatty acids and correlations between individual fatty acids and the LI. [file 12888_2023_5161_MOESM1_ESM.docx]

**Appendix 1**. Melting points of different fatty acids and correlations between individual fatty acids and the LI

| **Fatty acids** | **Melting point (°C)** | **Median Individual FA intake (g/day)** | **r^a^** |
| --- | --- | --- | --- |
| **SFA**  04:00  06:00  08:00  10:00  12:00  13:00  14:00  15:00  16:00  17:00  18:00  20:00  22:00  24:00:00 | -7.9  -3.4  16.7  31.6  44.2  41.5  53.9  52.3  63.1  61.3  69.6  76.75  81.5  88 | 0.4  0.3  0.3  0.8  0.9  0.0  3.0  0.0  12.2  0.0  6.0  0.0  0.0  0.0 | .362^**^  .369^**^  .307^**^  .367^**^  .248^**^  0.004  .401^**^  .040^*^  .073^**^  .042^*^  0.032  -.256^**^  -.258^**^  -.231^**^ |
| **MUFA**  14:01  15:01  16:01  17:01  18:01  20:01  22:01  24:01:00 | -4  4.5  0  57.5  16  [23.25](https://en.wikipedia.org/wiki/Gadoleic_acid)  [34.7](https://en.wikipedia.org/wiki/Erucic_acid)  [42.75](https://en.wikipedia.org/wiki/Nervonic_acid) | 0.1  0.0  1.1  0.0  25.4  0.1  0.0  0.0 | 0.006  0.011  .212^**^  -.042^*^  -.249^**^  -.260^**^  -0.034  -.169^**^ |
| **PUFA**  18:2 n-6  18:3  18:4  20:4 n-6  20:5 n-3  22:5 n-3  22:6 n-3 | [-5](https://en.wikipedia.org/wiki/Linoleic_acid)  [-11.15](https://en.wikipedia.org/wiki/Alpha-linolenic_acid)  [-57](https://en.wikipedia.org/w/index.php?title=Eicosadienoic_acid&action=edit&redlink=1)  [-49.5](https://en.wikipedia.org/wiki/Arachidonic_acid)  [-54.1](https://en.wikipedia.org/wiki/Eicosapentaenoic_acid)  [-78](https://en.wikipedia.org/wiki/Docosapentaenoic_acid)  -44.15 | 12.4  1.1  0.0  0.0  0.0  0.0  0.0 | -.515^**^  -.184^**^  -0.019  .103^**^  -.053^**^  -.054^**^  -.076^**^ |
| **TFA**  16:1 t  18:1 t n9  18:2 9t_12t | 31  45.5  28.5 | 0.0  0.9  0.0 | 0.019  .095^**^  -.145^**^ |

SFA saturated fatty acid, MUFA mono saturated fatty acid, PUFA poly saturated fatty acid, TFA trans fatty acid, ^a^The spearman’s correlation test is used for obtaining the results
